# Dataset for regulation between lncRNAs and their nearby protein-coding genes in human cancers

**DOI:** 10.1016/j.dib.2018.06.048

**Published:** 2018-06-26

**Authors:** Zhi Liu, Juncheng Dai, Hongbing Shen

**Affiliations:** a Department of Epidemiology and Biostatistics, Jiangsu Key Lab of cancer Biomarkers, Prevention and Treatment, Collaborative Innovation Center for cancer Medicine, School of Public Health, Nanjing Medical University, Nanjing, China; b Jiangsu Key Laboratory of Molecular and Translational cancer Research, Nanjing Medical University Affiliated cancer Hospital, Nanjing, China

**Keywords:** LncRNA, Cancer, Transcription factors

## Abstract

This article contains data related to the research article entitled “Systematic analysis reveals long noncoding RNAs regulating neighboring transcription factors in human cancers” (Liu et al., 2018 in press) [1]. Long noncoding RNAs (lncRNAs) are proposed to play essential roles in modulating the expression of the nearby loci. In this study, we systematically investigated the relationship between lncRNAs and their neighboring genes based on the genomic location of genes and the transcriptome expression profiles from TCGA samples across 12 tumor types. Position conservation analysis was applied to find lncRNAs conserved by position across vertebrate species. Gene ontology and enrichment analysis identified TF genes as a specific type of protein-coding genes that adjacent to highly positionally conserved lncRNA. The expression correlation of lncRNAs and their adjacent TFs were assessed across tumors to define significant co-expressed lncRNA-TF pairs, and a causal inference test (CIT) was used to infer the causal regulation of lncRNA on its nearby TF genes. A list of candidate lncRNA/TF regulation pairs in tumors was provided.

**Specifications Table**

**Table:** 

Subject area	*Biology*
More specific subject area	*Gene expression*
Type of data	*Tables*
How data was acquired	*Gene expression extracted from RNA-seq was downloaded from TANRIC and TCGA database.*
Data format	*Analyzed*
Experimental factors	*The expression of lncRNA and protein-coding genes were extracted from the total expression profiles.*
Experimental features	*Position conservation analysis was conducted on lncRNAs across ten vertebrate species to find lncRNAs conserved by position. Co-expression and causal inference test were used to infer causal relationship between lncRNA and their adjacent TF genes.*
Data source location	*N/A*
Data accessibility	*With this article*
Related research article	*Systematic analysis reveals long noncoding RNAs regulating neighboring transcription factors in human cancers. BBA molecular basis of disease. (In press)*

**Value of the data**

• The position conservation analysis of lncRNAs across species provides a reference for inferring the functionality of lncRNAs from the conservation perspective of view.
• The significant adjacency between positional conserved lncRNA and TF genes provides clues to study the regulation mechanism of lncRNAs on gene expression.
• The provided list of candidate lncRNA/TF regulation pairs can be used for experimental validation to investigate the function of lncRNA in tumors.

## 1 Data

### 1.1 GO enrichment of protein coding genes nearby lncRNA

GO items enriched by protein coding genes located in regions 1 Mb upstream and downstream lncRNA loci were presented in Table S1.

### 1.2 Position conservation of lncRNAs

The existence and absence of syntenic counterparts of human lncRNAs across other vertebrate species were listed in Table S2. LncRNAs that have syntenic lncRNAs in at least four species were classified as highly conserved ones (HC), and used in the following analysis. In total, 769 lncRNA/TF pairs were classified as HC pairs (Table S3). The detailed results were discribed [1].

### 1.3 Co-expression between lncRNA and TF genes

There were 266 of 769 HC lncRNA/TF pairs were significantly correlated in at least one tumor type, involving 159 TF genes and 253 lncRNAs (Table S4). Of those, 206 were consistently co-expressed in at least two tumor types.

### 1.4 Candidate lncRNA/TF regulation pairs

To prioritize the true lncRNA/TF regulatory pairs involved in tumors, we combined the results of co-expression (Table S4) and CIT (Table S5) and take advantage of pan-cancer dataset to define a confident list of pairs as those passed both co-expression test and CIT in more than two tumor types. Finally, we provided a list of 28 lncRNA/TF regulation pairs (Table 1).

**Table 1:** Candidate lncRNA/TF regulation pairs in TCGA tumors.

**lncRNA**	**TF genes**	**Tumor type**
SENCR	ETS1	BLCA,BRCA,HNSC,KIRC,LUAD,LUSC,OV,STAD
RP11-290F20.2	CEBPB	BLCA,BRCA,HNSC,KIRC,LUSC,STAD
RP11-290F20.1	CEBPB	BLCA,BRCA,HNSC,KIRC,LUSC,STAD
PVT1	MYC	BRCA,HNSC,KIRC,LUSC,OV,STAD
KB-1732A1.1	KLF10	BLCA,BRCA,HNSC,KIRC,LUSC,OV
AF064858.8	ETS2	HNSC,KIRC,LUAD,LUSC,OV
AF064858.11	ETS2	HNSC,KIRC,LUAD,LUSC,OV
RP11-796E10.1	SP3	HNSC,LUAD,LUSC,STAD
RP11-57H14.4	TCF7L2	BRCA,LUAD,LUSC,OV
RP11-290F20.3	CEBPB	BRCA,LUAD,LUSC,STAD
LINC00511	SOX9	BRCA,KIRC,LUAD,STAD
CASC15	SOX4	BRCA,KIRC,LUSC,STAD
RP6-109B7.4	PPARA	BRCA,KIRC,OV
RP11-57A1.1	SOX9	KIRC,LUAD,STAD
RP11-567M16.1	NFATC1	HNSC,LUSC,OV
RP11-51B23.3	TEAD1	BLCA,BRCA,LUAD
RP11-472N13.3	ZEB1	BLCA,BRCA,STAD
RP11-439L18.2	HIVEP2	BRCA,KIRC,STAD
RP11-397A16.2	TCF4	HNSC,KIRC,LUSC
RP11-330O11.3	ZEB1	BLCA,LUAD,LUSC
RP11-221N13.3	HMGA2	BLCA,HNSC,LUSC
PITRM1-AS1	KLF6	BRCA,KIRC,LUAD
LINC01152	SOX9	BRCA,LIHC,LUSC
LINC00261	FOXA2	LUSC,OV,STAD
GATA6-AS1	GATA6	LUAD,LUSC,STAD
CTD-2532K18.2	MSX2	HNSC,KIRC,LUSC
CCAT1	MYC	HNSC,LUSC,STAD

## 2 Experimental design, materials, and methods

### 2.1 Data and preprocessing

We downloaded TCGA lncRNA and coding gene expression data from the TANRIC database [2] (http://ibl.mdanderson.org/tanric/_design/basic/index.html) and Broad Institute GDAC firehose (http://gdac.broadinstitute.org) respectively. Only samples with paired lncRNA and mRNA expression profiles were used in this study. LncRNA with RPKM >0.1 and coding genes with RPKM >1 in at least 5% of the samples in each tumor types were retained for the following analysis (Table 2).

**Table 2:** The number of tumor samples and expressed lncRNAs across tumors.

**Tumor**	**No. of tumor samples**	**No. of expressed lncRNA**
BLCA	252	5979
BRCA	837	5941
COAD	157	1612
HNSC	426	5149
KIRC	448	6183
KIRP	198	5864
LIHC	200	4969
LUAD	488	6288
LUSC	220	6206
OV	412	6197
STAD	285	6070
THCA	497	5122

### 2.2 Positional conservation of human lncRNAs across species

Annotations of protein-coding gene orthologs were obtained from EnsemblCompara [3], and lncRNA annotation in other ten species was downloaded from the NONCODE database [4]. To identify syntenic human lncRNAs in other species, we used the method proposed by Hezroni et al. [5]. Briefly, when comparing genome human (H) and species A, and when considering orthologous protein-coding genes G1 and G2 we first identified lncRNAs within 5×105×Genome length(H)/10^9nt of G1 in H and within 5×105Genome length(A)/10^9nt of G2 in A. A lncRNA was considered to be found “upstream” of the protein-coding gene when it overlapped it or ended 5′ to its 5′ end, and “downstream” when it overlapped it or started 3′ to the 3′ end of the protein-coding gene. Two lncRNA L1 and L2 from A and B were considered syntenic, if they were both upstream or both downstream of G1 and G2, with the same relative orientations.

### 2.3 Co-expression between lncRNA and their nearby TF genes

Pearson correlation coefficient was used to analyze the co-expression between lncRNA and their nearby TF genes. Co-expressed gene pairs were identified with an absolute Pearson correlation coefficient value ≥0.25 and an FDR-adjusted *p*-value ≤0.05.

### 2.4 Causal inference analysis of lncRNA/TF regulation

The lncRNA-TF-targets regulation relationships were assessed using the causal inference test (CIT) [6] to test the regulation chain and to select the possible lncRNA-TF regulation pairs. Briefly, the CIT has statistical tests for four conditions, all of which must be met for the TF -mediated causal classification: (1) lncRNA and TF target are associated, (2) lncRNA is associated with T F after adjusting for TF target, (3) TF is associated with TF target after adjusting for lncRNA, and (4) lncRNA is independent of TF target after adjusting for TF. The CIT *p*-value was defined as the maximum of the component test *p* values, and a multivariate linear regression was used in the four component tests. The targets of each TF were obtained from the TRRUST database [7], which collect transcriptional regulatory relationships unraveled by sentence-based text-mining.
